# Uric acid is a strong independent predictor of renal dysfunction in patients with rheumatoid arthritis

**DOI:** 10.1186/ar2775

**Published:** 2009-07-24

**Authors:** Dimitrios Daoussis, Vasileios Panoulas, Tracey Toms, Holly John, Ioannis Antonopoulos, Peter Nightingale, Karen MJ Douglas, Rainer Klocke, George D Kitas

**Affiliations:** 1 Department of Rheumatology, Dudley Group of Hospitals NHS Trust, Russells Hall Hospital, Pensnett Road, Dudley, West Midlands, DY1 2HQ, UK; 2 Wolfson Laboratory, Department of Medical Statistics, School of Medicine, University of Birmingham, Queen Elizabeth Medical Centre, Edgbaston, Birmingham, B15 2TH, UK; 3 Arthritis Research Campaign Epidemiology Unit, University of Manchester, Oxford Road, Stopford Building, Manchester, M13 9PT, UK

## Abstract

**Introduction:**

Recent evidence suggests that uric acid (UA), regardless of crystal deposition, may play a direct pathogenic role in renal disease. We have shown that UA is an independent predictor of hypertension and cardiovascular disease (CVD), and that CVD risk factors associate with renal dysfunction, in patients with rheumatoid arthritis (RA). In this study we investigated whether UA associates with renal dysfunction in patients with RA and whether such an association is independent or mediated through other comorbidities or risk factors for renal impairment.

**Methods:**

Renal function was assessed in 350 consecutive RA patients by estimated glomerular filtration rate (GFR) using the six-variable Modification of Diet in Renal Disease equation. Risk factors for renal dysfunction were recorded or measured in all participants. Linear regression was used to test the independence of the association between GFR and UA.

**Results:**

Univariable analysis revealed significant associations between GFR and age, systolic blood pressure, total cholesterol, triglycerides, RA duration and UA. UA had the most powerful association with renal dysfunction (*r *= -0.45, *P *< 0.001). A basic model was created, incorporating all of the above parameters along with body mass index and gender. UA ranked as the first correlate of GFR (*P *< 0.001) followed by age. Adjustments for the use of medications (diuretics, low-dose aspirin, cyclooxygenase II inhibitors and nonsteroidal anti-inflammatory drugs) and further adjustment for markers of inflammation and insulin resistance did not change the results.

**Conclusions:**

UA is a strong correlate of renal dysfunction in RA patients. Further studies are needed to address the exact causes and clinical implications of this new finding. RA patients with elevated UA may require screening for renal dysfunction and appropriate management.

## Introduction

Renal dysfunction in patients with rheumatoid arthritis (RA) has been attributed to multiple factors, including the use of nephrotoxic medication, the presence of comorbitities such as hypertension and atherosclerosis and complications such as vasculitis or amyloidosis [1-3]. There has been recent epidemiologic and experimental evidence supporting the hypothesis that uric acid (UA), regardless of crystal deposition, may play a direct pathogenic role in multiple diseases, including renal disease [4,5].

UA is a ubiquitous by-product of purine metabolism and was thought to have a beneficial role by acting as an antioxidant [6]. Even though the link between impaired renal function and UA is well known, it has not received much attention, since hyperuricaemia was considered simply a consequence of decreased glomerular filtration rate (GFR). Recent evidence, however, supports the view that UA may not be just an innocent bystander but may be an active player in the pathogenesis of renal disease [7,8] by causing endothelial dysfunction [9], intrarenal vascular disease [10] and renal impairment [11]. The most compelling evidence comes from animal models in which induced hyperuricaemia in healthy rats caused renal cortical vasoconstriction and glomerular hypertension that was prevented by allopurinol treatment [12]. In rats with pre-existing renal disease, hyperuricaemia increased renal vascular damage [13]. A growing amount of evidence from prospective large-scale epidemiologic studies points to the direction of a strong link between UA and renal dysfunction in the general population. UA was shown to be a powerful independent predictor of prevalent renal dysfunction but was also a significant predictor of progression of renal disease [14-17]. In a recent meta-analysis of the prospective studies addressing the role of hyperuricaemia as a predictor of future renal disease among patients with normal GFR, conducted in the past 20 years, it was shown that most studies (eight out of nine) found that UA was an independent predictor [18].

We have previously shown that UA is an independent predictor of hypertension [19] and cardiovascular disease (CVD) [20] in patients with RA. We have also shown that renal dysfunction in RA is associated mainly with cardiovascular risk factors and not RA-related factors such as disease activity, severity or therapy [21]. In that study, UA was shown to associate with renal dysfunction in patients with RA. In this study, we focus on the potential association of UA with renal dysfunction in patients with RA and investigate whether such an association is independent or mediated through other comorbidities or risk factors for renal impairment. We aimed at exploring the hypothesis that UA might be the link between CVD and renal dysfunction in patients with RA. To the best of our knowledge, this is the first study that focuses on the role of UA in renal dysfunction in patients with RA.

## Materials and methods

### Participants

A cohort of 350 consecutive patients with RA meeting retrospective application of the 1987 revised American College of Rheumatology classification criteria [22] were recruited from routine outpatient clinics at the Department of Rheumatology of the Dudley Group of Hospitals, UK, for this cross-sectional, single-centre study. The study had local Research Ethics Committee and Research & Development Directorate approval, and all participants gave their written informed consent in accordance with the Declaration of Helsinki.

Basic demographic and clinical characteristics of the sample are shown in Table 1. The cohort consisted almost exclusively (96.0%) of people of white-British origin (reflecting the local demographic split) and most of them (71.7%) were female, as expected. Most participants (86.5%) were on disease-modifying antirheumatic drugs (DMARDs), with the most widely used being methotrexate (MTX). There were only two participants on cyclosporine, two on allopurinol and none on uricosuric therapy. No patients in this cohort were current users of either gold or penicillamine, but a limited number (17 for gold and 33 for penicillamine) had used these agents in the past. All participants underwent a thorough baseline evaluation, including a review of their medical history and hospital records, physical examination (including height, weight and body mass index [BMI]), calculation of current disease activity score using 28 joint counts (DAS28) [23] and self-report of current functional disability on the anglicised Health Assessment Questionnaire (HAQ) [24]. All medications, including low-dose aspirin, diuretics, nonsteroidal anti-inflammatory drugs (NSAIDs) and cyclooxygenase II (COX-II) inhibitors, were recorded. Venous blood was collected in the fasting state on the day of baseline assessment, and relevant tests were performed. All tests were performed in one laboratory at the Dudley Group of Hospitals. Renal function was assessed by GFR estimation using three different predictive equations: the six-variable Modification of Diet in Renal Disease (MDRD) equation [25], the abbreviated MDRD formula [26] and the classic Cockcroft-Gault formula [27]. GFR estimates presented here are based only on the six-variable MDRD equation, estimated GFR = 170 × (creatinine)^-0.999 ^× (age)^-0.176 ^× (serum urea nitrogen)^-0.170 ^× (albumin)^+0.318 ^× (0.762 if the person is female), since there were no differences in the pattern of significance of findings arising from the full data analysis based on any of the three formulae. Traditional risk factors for renal dysfunction were recorded/assessed in all patients. Blood pressure (BP) was the mean of three measurements taken from the left arm with the patient seated. The presence of hypertension was defined as a systolic BP of greater than 140 and/or diastolic BP of greater than 90 mm Hg and/or the use of antihypertensive medications [28]. Patients were defined as being diabetic when fasting serum glucose levels were greater than 7 mmol/L and/or oral hypoglycaemic medications or insulin was used [29]. The number of pack-years of smoking was recorded, and patients were also separated into three groups: current smokers, ex-smokers and never smoked. Alcohol consumption was recorded as the number of units consumed per week in those patients who admitted to drinking more than the maximum recommended weekly levels of 21 and 14 units for males and females, respectively. Biochemical estimations included fasting lipids, complete serum biochemistry (including UA), fasting glucose, fasting insulin and C-reactive protein (CRP). Reference ranges for UA were established in our Clinical Pathology Accreditation-accredited laboratory based on the mean ± 2 standard deviations (SDs) of samples of apparently healthy adult males and females from the local population (data on file). Insulin resistance was evaluated from fasting glucose and insulin using the Homeostasis Model Assessment of Insulin Resistance (HOMA IR) [30] and the Quantitative Insulin Sensitivity Check Index (QUICKI) [31].

**Table 1:** Characteristics of the patients with rheumatoid arthritis (n = 350)

Characteristics		*P *value
Age in years, mean ± SD	61.8 ± 11.8	< 0.001^a^

Female gender, number (percentage)	251 (71.7)	NS^b^

White-British race, number (percentage)	336 (96)	NS^b^

RF-positive, number (percentage)	238 (68)	NS^b^

Smoking status, number (percentage)		
Never smoked	157 (44.8)	NS
Ex-smoker	133 (38)	
Current smoker	60 (17.2)	

Pack-years, median (quartiles)	13.5 (5–30)	NS^c^

BMI in kg/m^2^, mean ± SD	27.1 ± 5.1	NS^a^

RA duration in years, median (quartiles)	10 (4–19)	0.01^c^

Disease activity (DAS28), mean ± SD	4.2 ± 1.37	NS^a^

CRP in mg/L, median (quartiles)	16.8 (5–20.7)	NS^c^

Hypertension, number (percentage)	256 (73.1)	0.008^b^

Systolic BP in mm Hg, mean ± SD	143.4 ± 20.9	0.002^a^

Diastolic BP in mm Hg, mean ± SD	78.9 ± 11.4	NS^a^

Total CHOL in mmol/L, mean ± SD	5.2 ± 1.2	0.018^a^

HDL CHOL in mmol/L, mean ± SD	1.6 ± 0.45	NS^a^

TG in mmol/L, median (quartiles)	1.43 (1–1.6)	0.001^c^

Diabetes mellitus, number (percentage)	25 (7.1)	NS^b^

HOMA IR, median (quartiles)	1.95 (1.2–3.3)	0.05^c^

QUICKI, mean ± SD	0.34 ± 0.04	0.05^a^

DMARDs, number (percentage)	303 (86.5)	NS^b^

MTX, number (percentage)	196 (56)	NS^b^

MTX dose in mg, median (quartiles)	10 (7.5–18.7)	NS^c^

Prednisolone, number (percentage)	110 (31.4)	NS^b^

Prednisolone dose in mg, median (quartiles)	7.5 (4.3–10)	NS^c^

NSAIDs, number (percentage)	68 (19.4)	NS^b^

COX-II inhibitors, number (percentage)	31 (8.8)	NS^b^

ACE inhibitors, number (percentage)	91 (26)	0.001^b^

Diuretics, number (percentage)	87 (24.8)	0.02^b^

Aspirin, number (percentage)	57 (16.2)	NS^b^

*P *values represent the significance of the association with glomerular filtration rate. The symbols after *P *values show the corresponding statistical test: ^a^for Pearson correlation, ^b^for *t *test and ^c^for Spearman correlation. ACE: angiotensin-converting enzyme; BMI: body mass index; BP: blood pressure; CHOL: cholesterol; COX-II: cycloxygenase II; CRP: C-reactive protein; DAS28: disease activity score using 28 joint counts; DMARD: disease-modifying antirheumatic drug; HDL: high-density lipoprotein; HOMA IR: homeostasis model assessment of insulin resistance; MTX: methotrexate; NS: nonsignificant; NSAID: nonsteroidal anti-inflammatory drug; QUICKI: quantitative insulin sensitivity check index; RA: rheumatoid arthritis; RF: rheumatoid factor; SD: standard deviation; TG: triglycerides.

### Statistical analysis

Statistical analyses were performed using SPSS, version 13.0 (SPSS Inc., Chicago, IL, USA). Variables were tested for normality by applying the Kolmogorov-Smirnov test. Data are presented as mean ± SD, median (upper and lower quartile values), or percentages, as appropriate. Relationships between GFR (continuous variable) and other variables were analysed by *t *tests and Pearson or Spearman correlations, as appropriate. Linear regression was used to test the independence of the association between GFR and UA.

## Results

The mean ± SD of the GFR in the whole sample was 82.16 ± 21.50 mL/min/1.73 m^2^. One hundred sixteen participants (33%) had a normal GFR of greater than 90 mL/min/1.73 m^2^, 185 participants (53%) had mild renal impairment with GFR of 60 to 90 mL/min/1.73 m^2 ^and 49 participants (13%) had moderate renal impairment with GFR of less than 60 mL/min/1.73 m^2^. No patients in this cohort had a GFR of less than 30 mL/min/1.73 m^2 ^to suggest severe renal impairment. Mean ± SD of UA was 310.9 ± 90.6 μmol/L: only 31 participants (6 men and 25 women) were hyperuricaemic as defined by UA levels of greater than 500 μmol/L for men and of greater than 400 μmol/L for women.

**Figure 1:**
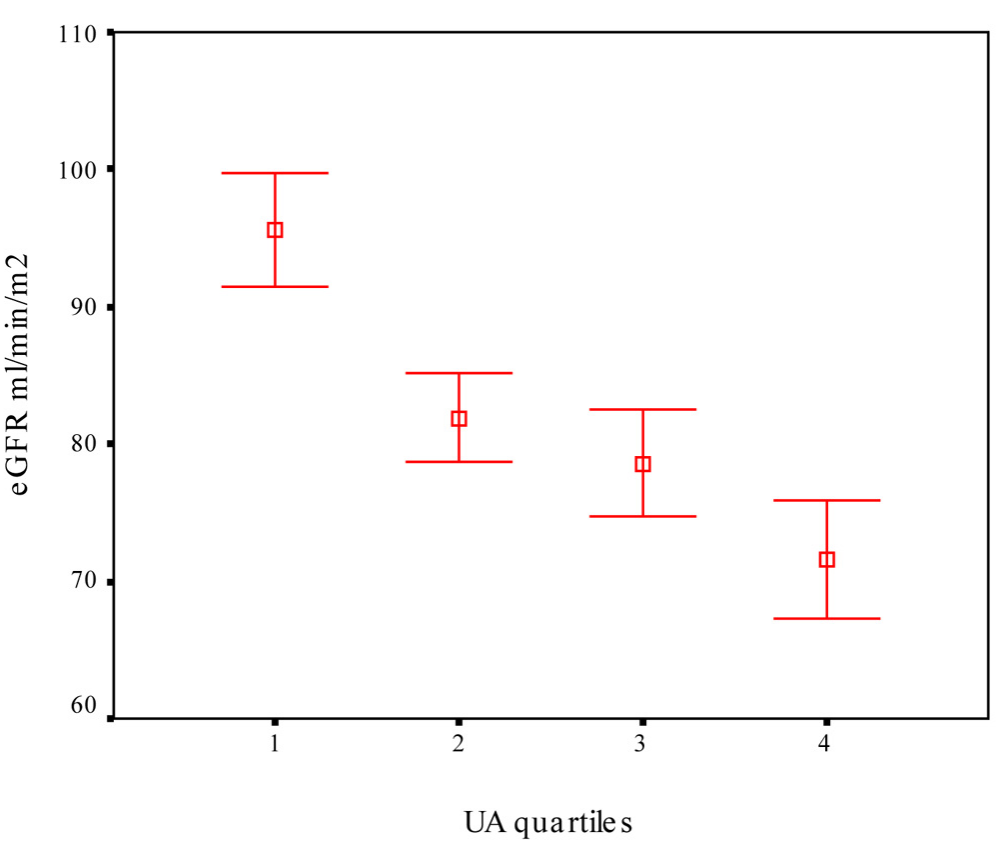
Mean ± standard error of the mean of estimated glomerular filtration rate (eGFR) stratified according to uric acid (UA) quartiles. A roughly linear inverse association can be seen (1 = lowest, 4 = highest quartile).

In univariable analysis, UA was strongly inversely associated with GFR (*r *= -0.45, *P *< 0.001). This was the strongest association found between GFR and any of the other variables studied, including age (*r *= -0.44, *P *< 0.001), despite the fact that age is considered the most powerful predictor of renal function and is included in all GFR predictive equations. On splitting the population on quartiles based on UA levels, a roughly linear inverse association of GFR with UA was observed (Figure 1). The values of mean ± SD of the GFR in the quartiles were 95.57 ± 20.8, 81.89 ± 16.19, 78.62 ± 19.2 and 71.63 ± 21.36 mL/min/1.73 m^2 ^from the lowest to the highest UA quartile, respectively. Other variables found to have significant associations with GFR were RA duration, the presence of hypertension, systolic BP, total cholesterol (TCHOL), triglycerides (TG), insulin resistance either by HOMA IR or QUICKI, the use of angiotensin-converting enzyme inhibitors and diuretics (Table 1). The above-mentioned variables, apart from QUICKI, were inversely associated with GFR. We found no associations with gender, BMI, smoking status or pack-years, disease activity (DAS28, erythrocyte sedimentation rate and CRP), functional disability (HAQ), high-density lipoprotein cholesterol, presence of diabetes, use of any DMARD (or MTX specifically) either currently or in the past, NSAIDs, COX-II inhibitors or steroids.

We further collected data regarding conditions that may be associated with hyperuricaemia (alcohol consumption, psoriasis, thyroid disease and use of low-dose aspirin). Less than 2% of the total number of patients assessed in this study admitted to drinking more than the recommended maximum weekly levels of 21 and 14 units for males and females, respectively; none of them was hyperuricaemic. This is why we have not included alcohol consumption in the univariable analysis. None of the participants had psoriasis, and less than 5% had thyroid-stimulating hormone levels outside normal limits.

The independence of the strong association between UA and GFR was evaluated using linear regression. An initial model (model 1) was created incorporating all of the risk factors for renal impairment found to contribute significantly from the univariable analysis (age, systolic BP, TCHOL, TG, disease duration and UA) as well as gender and BMI, which related to many of the variables (Table 2). UA was the strongest correlate of GFR (β = -0.45, *P *< 0.001), followed by age (β = -0.41, *P *< 0.001). Results were similar when the presence of hypertension was entered instead of continuous systolic BP. UA remained the strongest predictor (β = -0.47, *P *< 0.001) when the use of diuretics, low-dose aspirin, COX-II inhibitors and NSAIDs were also included in the model. The strong association between UA and GFR was not reduced (β = -0.48, *P *< 0.001) by further adjustments for inflammation (by including CRP) and insulin resistance (by including HOMA IR) to the previous model or repeat multivariable regression analysis with a stepwise and backward procedure.

**Table 2:** Multivariate analysis

**Model 1**			
	Coefficient β	Standard error (β)	*P *value
Uric acid	-0.450	0.011	< 0.001
Age	-0.410	0.082	< 0.001
Female gender	-0.175	0.025	0.003
Total cholesterol	-0.122	0.031	0.025
Triglycerides	-0.019	0.016	NS
Systolic blood pressure	-0.043	0.041	NS
Body mass index	-0.044	0.043	NS
Rheumatoid arthritis duration	-0.028	0.021	NS

NS: non-significant.

To evaluate whether the predictive value of UA was retained when its levels were well within the normal range, we repeated the analysis after exclusion of participants with UA of greater than 400 μmol/L (n = 56) and the results were similar (β = -0.27, *P *< 0.001, controlling for all of the potential confounders mentioned above). Similar results were obtained when a different threshold for the definition of hyperuricaemia was used: 416 μmol/L (7 mg/dL) and 357 μmol/L (6 mg/dL) for males and females, respectively (data not shown). However, when the analysis was repeated only in participants with normal renal function (GFR of greater than 90 mL/min/1.73 m^2^) (n = 116), UA was no longer associated with GFR in either the univariable (*r *= 0.11, *P *= 0.24) or multivariable (β = -0.06, *P *= 0.61) analysis, suggesting that UA is not a predictor of GFR in such cases.

## Discussion

The present cross-sectional study has shown that UA, irrespective of the presence of hyper- or normo-uricaemia, was the strongest independent predictor of GFR in patients with RA, even after adjustments for most of the potential confounding factors. This association was not present in patients with normal renal function.

GFR in the present study was not assessed by direct measurement and this is a potential limitation. Radioisotope methods with the use of Cr-EDTA (chromium-ethylenediaminetetraacetic acid) are considered the gold standard for direct GFR measurement but are expensive, time-consuming and not easily applied in large cohorts such as this. Conversely, 24-hour urine collections for determining creatinine clearance are inaccurate and are being abandoned. Estimated GFR from predictive equations are generally accurate and have been validated in very large cohorts [32]. Specifically with respect to RA patients, predictive equations have shown very good correlation with direct GFR measurements, despite the initial concerns that muscle wasting, a common feature of RA, could lead to overestimation of GFR [33,34]. The presented results were reproduced when the classic Cockcroft-Gault or the abbreviated MDRD formula was used (data not shown) and this consistency enhances their strength.

The association of UA levels with renal dysfunction in the general population is well known but was attributed solely to the fact that UA is excreted mainly through the kidneys and a decline in GFR increases its level. However, patients with even severe renal impairment have only minimal hyperuricaemia due to a significant compensatory increase in gastrointestinal excretion [35]. Our study suggests that such an association also occurs in patients with RA. Even if this simply reflects a decline in glomerular function, serial measurement of UA could serve as a biomarker for the early detection of subtle changes in the glomerular function of patients with RA and help identify patients at risk of developing renal impairment.

However, recent experimental, epidemiologic and clinical studies suggest that UA may contribute directly or indirectly to the pathogenesis of renal disease. Most of the evidence for a direct pathogenic role comes from animal studies in healthy rats in which mild hyperuricaemia, without crystal deposition, was induced with the use of the uricase inhibitor oxonic acid. This resulted in the development of interstitial renal injury and hypertension, both of which were prevented by the use of allopurinol [12]. Further studies in this rat model have demonstrated the occurrence of renal vascular changes, including afferent arteriolopathy with thickening and hypercellularity that occurred independently from changes in BP [36] or glomerular hypertension and hypertrophy [37]. These vascular changes were considered a consequence of direct stimulation of vascular smooth muscle cells (VSMCs) by UA. Indeed, UA has been shown to stimulate VSMC proliferation *in vitro *by activating the mitogen-activated protein kinase and extracellular-regulated kinase (ERK 1/2) and upregulating platelet-derived growth factor and its receptor [38].

Clinical studies also suggest an association of UA with renal dysfunction. A large-scale study of 6,400 people from the general population with normal renal function revealed that UA was a powerful and independent predictor for developing renal impairment in 2 years [39]. In another prospective study addressing the prevalence and predictors of renal impairment in the general population, UA scored as the second strongest independent risk factor for renal impairment after hypertension [40]. The significant role of UA in the progression of renal disease was also underscored in a recent very large study that included more than 175,000 individuals in a 25-year follow-up, in which UA was shown to be an independent predictor of end-stage renal disease [17]. Based on such data, the first trial of allopurinol in chronic kidney disease has been conducted in a small cohort of patients and suggests that such treatment aids preservation of renal function during the 12 months of therapy compared with controls [41]. On the other hand, in a cohort of patients with chronic kidney disease treated with allopurinol, discontinuation of allopurinol led to a significant acceleration of the rate of loss of kidney function [42].

The above studies highlight the role of UA as an independent predictor of renal dysfunction in the general population. The present study provides evidence that this is also the case for patients with RA. It is worth noticing, however, that the link between UA and renal dysfunction as depicted in the present study is stronger than that reported in the general population. In patients with RA, UA scored as the strongest predictor of renal dysfunction; this was not observed in the epidemiologic studies in the general population, in which more traditional risk factors for renal dysfunction such as proteinuria and obesity were reported to be stronger predictors of renal impairment than UA [17]. This may partially relate to the higher prevalence of CVD [43] and the metabolic syndrome [44] in patients with RA, both of which are tightly linked to hyperuricaemia [20,45].

Apart from a direct pathogenic association of UA with renal dysfunction, alternative explanations may also apply. To start with, UA may just be 'marking' patients with increased cardiovascular or renal risk [46]. Hyperuricaemia has been shown to predict the development of CVD in the general population [47,48] and in subjects with hypertension [49,50] or pre-existing CVD [51]. With respect to RA patients, we have previously shown that UA is an independent predictor of CVD [20]. Hypertension may be another strong potential link between UA and renal dysfunction: induced hyperuricaemia in healthy rats causes hypertension and salt sensitivity [52], whereas in humans, childhood serum urate levels predict higher adult BP independent of childhood BMI [53]. Hypertensive adolescents have a higher prevalence of hyperuricaemia, and lowering of UA is accompanied by BP reduction [54]. Again, hypertension is highly prevalent in patients with RA [55,56] and associates with hyperuricaemia as well [19]. Vascular disease mediated through endothelial dysfunction may be another link. The role of UA as a mediator of endothelial dysfunction by nitric oxide (NO) inactivation has recently emerged [57,58]. The xanthine oxidase system is one of the main producers of superoxide radicals in vascular endothelium and therefore UA could be a mediator of vascular disease that could potentially lead to renal impairment. Abnormalities in NO-dependent vasodilation in patients with RA are well described and are thought to be an early marker of accelerated atherosclerotic disease [59]. Finally, yet another indirect link may be insulin resistance-metabolic syndrome. It has been proposed that hyperinsulinemia stimulates UA reabsorption in the proximal tubule [60]. There is evidence correlating the metabolic syndrome with impaired renal function, even in nondiabetic subjects [61-63]. A recent large-scale study identified a positive strong association between insulin resistance and chronic kidney disease in nondiabetic patients, independent of other risk factors [64]. Insulin resistance has also been described in patients with RA and may associate with systemic inflammation [44].

In the present study, we had the opportunity to collect contemporary data relevant to most of the above potential links and made all of the required adjustments in the multivariable analysis, although residual confounding cannot be excluded. For example, socioeconomic status, which has also been linked to renal dysfunction [65] as well as RA [66], was not assessed in this cohort. Several of the comorbidities assessed here, including hypertension and insulin resistance, showed a clear association with renal impairment in this cohort of RA patients. However, the fact that UA was the strongest predictor and was independent from all the traditional risk factors for cardiovascular or renal disease suggests that, in this population, UA may indeed play a direct pathogenic role in the development of renal dysfunction. Taking into consideration that UA associates with both hypertension and CVD, this study provides indirect evidence that UA might be the link between CVD and renal dysfunction in RA. Due to the cross-sectional nature of our study, this interpretation can be made only with great caution and prospective studies are needed before any definite conclusions are drawn.

## Conclusions

In summary, this study shows that UA is a powerful independent predictor of renal dysfunction in patients with RA. Its possible direct pathogenic role and potential clinical use as an early biomarker of future renal dysfunction in this group of patients need to be investigated in prospective studies designed specifically for the purpose.

## Abbreviations

BMI: body mass index; BP: blood pressure; COX-II: cyclooxygenase II; CRP: C-reactive protein; CVD: cardiovascular disease; DAS28: disease activity score using 28 joint counts; DMARD: disease-modifying antirheumatic drug; GFR: glomerular filtration rate; HAQ: health assessment questionnaire; HOMA IR: homeostasis model assessment of insulin resistance; MDRD: modification of diet in renal disease; MTX: methotrexate; NO: nitric oxide; NSAID: nonsteroidal anti-inflammatory drug; QUICKI: quantitative insulin sensitivity check index; RA: rheumatoid arthritis; SD: standard deviation; TCHOL: total cholesterol; TG: triglycerides; UA: uric acid; VSMC: vascular smooth muscle cell.

## Competing interests

The authors declare that they have no competing interests.

## Authors' contributions

DD carried out the analysis and interpretation of data, drafted the manuscript and participated in data acquisition. VP participated in data acquisition, provided technical assistance and assisted in analysis and interpretation of data. TT, HJ and IA participated in data acquisition. PN performed the statistical analysis and assisted in manuscript preparation. KMJD and RK participated in data acquisition and assisted in manuscript preparation. GDK conceived the idea of the study and assisted in manuscript preparation. All authors read and approved the final manuscript.
